# Hox, Wnt, and the evolution of the primary body axis: insights from the early-divergent phyla

**DOI:** 10.1186/1745-6150-2-37

**Published:** 2007-12-13

**Authors:** Joseph F Ryan, Andreas D Baxevanis

**Affiliations:** 1 Genome Technology Branch, National Human Genome Research Institute, National Institutes of Health, Bethesda, MD 20892, USA

## Abstract

The subkingdom Bilateria encompasses the overwhelming majority of animals, including all but four early-branching phyla: Porifera, Ctenophora, Placozoa, and Cnidaria. On average, these early-branching phyla have fewer cell types, tissues, and organs, and are considered to be significantly less specialized along their primary body axis. As such, they present an attractive outgroup from which to investigate how evolutionary changes in the genetic toolkit may have contributed to the emergence of the complex animal body plans of the Bilateria. This review offers an up-to-date glimpse of genome-scale comparisons between bilaterians and these early-diverging taxa. Specifically, we examine these data in the context of how they may explain the evolutionary development of primary body axes and axial symmetry across the Metazoa. Next, we re-evaluate the validity and evolutionary genomic relevance of the zootype hypothesis, which defines an animal by a specific spatial pattern of gene expression. Finally, we extend the hypothesis that Wnt genes may be the earliest primary body axis patterning mechanism by suggesting that Hox genes were co-opted into this patterning network prior to the last common ancestor of cnidarians and bilaterians.

Reviewed by Pierre Pontarotti, Gáspár Jékely, and L Aravind. For the full reviews, please go to the Reviewers' comments section.

## Background

**Figure 1:**
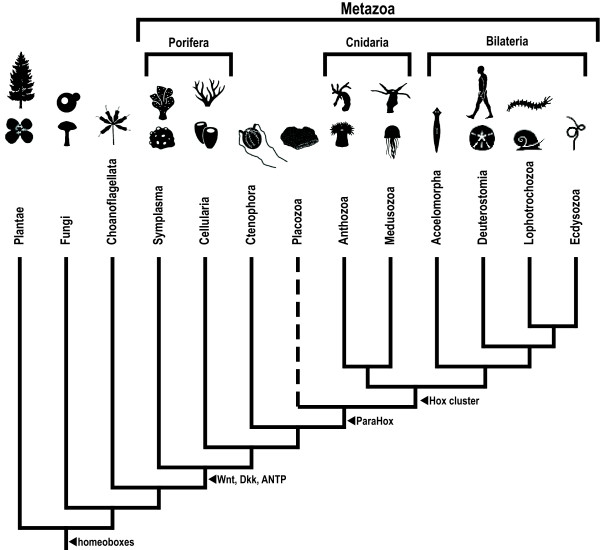
Evolutionary relationships of early-diverging metazoan lineages (Symplasma, Cellularia, Ctenophora, Placozoa, Anthozoa, and Medusozoa) with Bilateria and outgroups (Plantae, Fungi, and Choanoflagellata). Tree topology is based on [77]. Dotted line indicates uncertainty as to the placement of the placozoan branch. Arrows indicate the earliest known appearance of the specified group of genes.

The thirty-plus metazoan phyla are each characterized by a distinct "Bauplan" (or body plan; see [1,2]). A major challenge facing evolutionary biologists lies in understanding the evolution of major animal features such as body symmetry, germ layers, body cavities, skeletal systems, and nervous systems that comprise these disparate metazoan body plans [3]. In approaching these questions, it is especially useful to consider the earliest-branching metazoan phyla – specifically, Porifera (sponges), Ctenophora (ctenophores), Placozoa (*Trichoplax*), and Cnidaria (*e.g*., sea anemones, corals, and jellyfish) (Figure 1). The origin of animals dates back to over 600 million years ago and perhaps much earlier [4]. Fossil evidence and molecular evidence suggest bilaterian animals radiated during the Cambrian period some 500–550 million years ago [5]. In the interval between these two events, the Symplasma and the Cellularia (both sub-phyla within Porifera), the Ctenophora, the Cnidaria, and the Placozoa emerged in succession as independent lineages (see Figure 1; see also Table 1 and references therein). Each of these early branching lineages offers a unique perspective into early animal evolution.

**Table 1:** Summary of results from ribosomal phylogenetic analyses. Only studies that included sequences from at least one species of Porifera, Ctenophora, Placozoa, Cnidaria, and Bilateria were considered. Not all the studies included data from both major clades of poriferans, so a single "Po" entry in the result column does not necessarily indicate a monophyletic Porifera. Modeled after Table 1 of [99].

**Authors**	**Year**	**Meth**	**Result**	**Hypoth**
Wainright et al. [79]	1993	ML	(Po,(Ct,((Tr, Cn), Bi)))	
Katayama et al. [80]	1995	DI	((((Po, Ct), Tr), Cn), Bi)	
Katayama et al. [80]		MP	((((Po, Ct), Tr), Cn), Bi)	
Katayama et al. [80]		ML	((Po,(Ct, Tr)),(Cn, Bi))	CnBi
Hanelt et al. [81]	1996	DI	((((Po, Ct), Tr), Cn), Bi)	
Van de Peer & Wachter [82]	1997	DI	(((Po,(Po, Ct)),(Tr, Cn)), Bi)	
Abouheif et al. [83]	1998	MP	(Po,(Ct,(Tr,(Cn, Bi))))	CnBi
Colins [84]	1998	MP	(Po,(Po, Ct,(Tr,(Cn, Bi)))	CnBi
Colins [84]		ML	(Po,((Po, Ct),(Tr,(Cn, Bi))))	CnBi
Halanych [85]	1998	MP	(Po,(Tr,((Cn,(Ct, Cn)), Bi)));	
Halanych [85]		MP	(Po,(Tr,(Ct, Cn, Bi))))	
Lipscomb et al. [86]	1998	MP	(Po, Po, Ct,((Tr, Cn), Bi));	
Littlewood et al. [87]	1998	DI	(Tr,((Po,(Po, Ct)),(Cn, Bi)))*	CnBi
Winnepenninckx et al. [88]	1998	DI	((Po,(Po, Ct)),(Cn,(Tr, Bi)))	TrBi
Zrzavý et al. [89]	1998	MP	((Po,(Po, Ct)),(Tr,(Cn,(Cn, Bi))))	CnBi
Kim et al. [90]	1999	DI	(Po,(Po,(Ct,(Tr,(Cn, Bi)))))	CnBi
Kim et al. [90]		ML	(Po,(Po,(Ct,(Tr,(Cn, Bi)))))	CnBi
Giribet et al. [91]	1999	MP	(Po,(Ct,(Cn,(Tr, Bi))))	TrBi
Siddall & Whiting [92]	1999	MP	((Po, Ct),(Cn,(Tr, Bi)))	TrBi
Peterson & Eernisse [93]	2001	MP	(Po,(Po, Ct,(Tr,(Cn, Bi))))	CnBi
Podar et al. [94]	2001	ML	(Po,(Ct,(Tr,(Cn, Bi))))	CnBi
Collins et al. [95]	2002	MP	(Po,(Po,(Ct,(Cn,(Tr, Bi)))))	TrBi
Jondelius et al. [96]	2002	ML	(Po,(Po,(Ct,(Cn,(Tr, Bi)))))	TrBi
Martinelli & Spring [97]	2003	ML	(Po,(((Ct, Tr), Cn), Bi))	
Zrzavý & Hypša [98]	2003	MP	(Po,(Ct,((Tr, Cn), Bi)))	
Zrzavý & Hypša [98]		MP	(Po,(Ct,(Tr,(Cn, Bi))))	CnBi
Zrzavý & Hypša [98]		MP	(Po,((Ct, Tr),(Cn, Bi)))	CnBi
Wallberg et al. [99]	2004	MP	(Po, Po,(Ct,(Cn,(Tr, Bi))))	TrBi

Meth = Method, Hypoth = Hypothesis, ML = Maximum Likelihood, MP = Maximum Parsimony, DI = Distance, Po = Porifera, Ct = Ctenophora, Tr = Placozoa, Cn = Cnidaria, Bi = Bilateria, CnBi = sister relationship between Cnidaria and Bilateria, TrBi = sister relationship between Placozoa and Bilateria.

One key morphological feature that has contributed to the traditional super-phyletic relationships of animals is symmetry about a primary body axis. A comprehensive analysis of animal anatomy produced by Beklemishev concluded that all animals are organized by a primary body axis (alternatively referred to as "anterior/posterior", "apical/basal", and "oral/aboral") [6]. This axis is especially evident during embryogenesis and larval stages. It is debated though whether this monaxonal symmetry is homologous throughout the Metazoa and at what point (or points) in animal evolution bilateral symmetry emerged.

### Axial symmetry and the porifera

**Figure 2:**
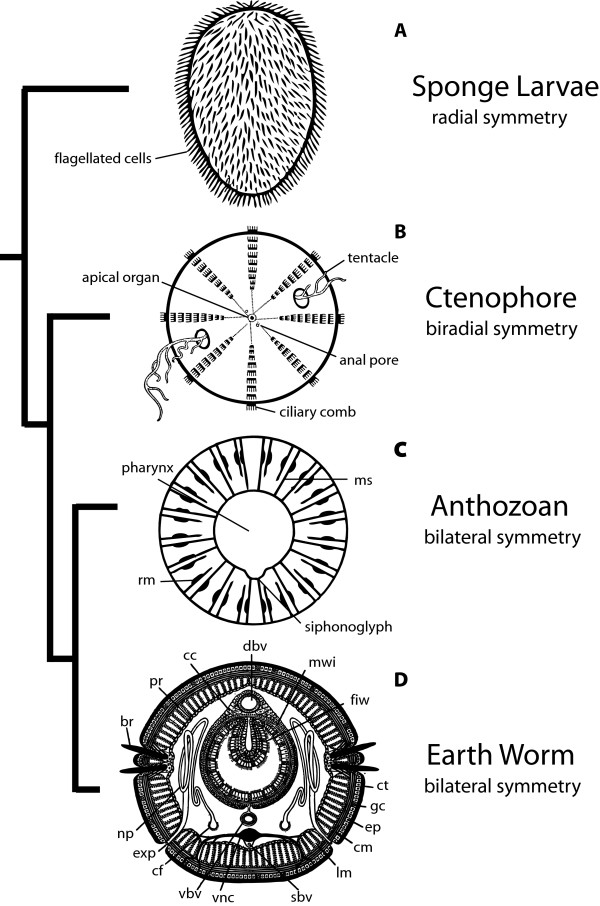
Phylogenetic framework of primary body axis symmetry. (A) Radial symmetry of a sponge larva. (B) Top down view of *Pleurobrachia *(Ctenophora). The tentacles and anal pores of the ctenophore disrupt the octoradiate symmetry established by the 8 ciliary combs. (C) Bilateral symmetry of *Actinia *as seen through a cross-section of Actinia. The decemradiate symmetry defined by the septa is broken by a single siphonoglyph. (D) Bilateral symmetry defined by numerous structures as seen through the Cross-section of an earthworm. Abbreviations: ms = mesenteries, rm = retractor muscle, dbv = dorsal blood vessel, mwi = muscular wall of intestine, fiw = fold of intestinal wall, ct = cuticle, gc = gland cell, ep = epidermis, cm = circular muscles, lm = longitudinal muscles, sbv = subneural blood vessel, vnc = ventral nerve chord, vbv = ventral blood vessel, cf = ciliated funnel, exp = excretory pore, np = nephridium, br = bristle (only two of the 4 pairs of bristles are shown), pr = peritoneum, cc = chloragen cells. Illustrations in A, B, and D are after [78] with permission from University of Chicago Press. Illustration in C after [6].

Sponges are often described as being asymmetric or containing imperfect radial symmetry [6]. While it is true that adult sponges are often asymmetrically shaped by their environment, sponge larvae are usually symmetrical about their primary body axis, especially in the Hexactinelida and Calcarea classes [6,7] (Figure 2A). Moreover, many of the hallmark features of metazoan ontogeny (that is, cleavage, blastulation, and gastrulation) occur in sponges [8]. This fact is of critical importance when trying to reconstruct the body plan of the crown ancestor to metazoans. Recent reports have revealed that the phylum Porifera is genetically more complex than was anticipated [9-11]. They possess many of the signaling pathways and transcription factors that are involved in patterning the axes of bilaterians. There has yet to be extensive *in situ *hybridization assays involving sponges, but early results from the demosponge *Amphimedon queenslandica *suggest that transcription factors have the potential to specify distinct regions of the developing sponge [11]. Further insight into the processes responsible for patterning the sponge primary body axis will be essential in understanding the evolution of animal body plans.

### Axial symmetry and the "radiata"

The superphyletic designation Radiata was originally proposed by Cuvier to encompass the so-called radially-symmetric animals (for example, jellyfish, polyps, starfish, sea urchins, and some Protozoa) [12]. As new evidence has surfaced, the composition of this group has been modified several times (c.f. [13,14]). The term Radiata has also been frequently used to describe only the phyla Cnidaria and Ctenophora (for example, [15]). The rotating membership and the polyphyletic nature of these phyla (see Figure 1) contribute to the obvious confusion surrounding this term. However, the most confounding aspect of the term Radiata is that few species within Cnidaria and Ctenophora (the two consistent members of this group) actually exhibit true radial symmetry (that is, indefinite multiradiate symmetry), with many species within the group (particularly anthozoans) instead exhibiting bilateral symmetry.

Multiple examples of asymmetry, bilaterality, biradiality, and tetraradiality can be found collectively within the Cnidaria and Ctenophora (reviewed in [3,15-17]). In particular, many species within the Anthozoa (representing all three subclasses) and several within the Hydrozoa show clear signs of bilateral symmetry [6,18]. It is possible that this bilateral symmetry derived independently within these lineages, as suggested by Hyman and Beklemishev [6,19]. However, these authors were basing their conclusions on an animal phylogeny that placed the biradial ctenophores sister to the Bilateria. Furthermore, their understanding of cnidarian evolution was substantially different. Evidence based on both genomic content [20] and mitochondrial structure [21] suggest that the anthozoan biphasic lifestyle may be more primitive than medusozoans; this is in contrast to the more popular view at that time, a view put forth by Hyman that advocated the reverse scenario [19]. This recent shift in phylogenetic perspective supports the *turbellarian theory*, developed by Hadzi [22] and advocated by both Hand [23] and Willmer [3]: that radial symmetry is a derived feature within the Cnidaria, and suggests that bilateral symmetry evolved prior to the cnidarian-bilaterian ancestor. Data from the sea anemone *Nematostella vectensis *show that similar molecular mechanisms, including Hox, Dpp and Wnt genes [24-28], are involved in patterning the primary and secondary axes of bilaterians and cnidarians further suggesting that bilateral symmetry is plesiomorphic with respect to the Bilateria.

### Graded morphological complexity

It has been recognized previously that in terms of cell types and tissue types along the primary body axis, sponges are much simpler than bilaterians (i.e., sponges lack true tissues and organs) and that cnidarians occupy an intermediate level of axial complexity [29]. If the bilateral symmetry exhibited in anthozoans is considered the ancestral cnidarian condition, a pattern emerges of increasing morphological complexity along the primary body axis at each phyletic divergence point leading to the last common ancestor of bilaterians (Figure 2). Because of its uncertain phylogenetic position, *Trichoplax *is not considered in this model. Still, Placozoa is either the earliest diverging metazoan lineage (see [30]), in which case its asymmetry appropriately fits the model, or it is secondarily reduced (see Table 1 and references therein), in which case its asymmetry would not be appropriate to consider in this context. Sponges lack the complex organs and organ systems that are found in other Metazoa and are instead comprised of a series of asymmetric water current channels that define their (often imperfect) radial symmetry. The biradial nature of ctenophores is defined by the pharynx, the gastrovascular canals, and two sets of diametrically opposed structures: the anal pores and tentacles (Figure 2B). These structures break up the underlying multiradiate symmetry of these animals. Most bilaterally symmetrical anthozoans have underlying biradiate symmetry, defined by the pharynx and mesenteries; this symmetry is disrupted by a single structure, the siphonoglyph (and sometimes, the arrangement of the retractor muscles that attach to the mesenteries), which defines their bilateral symmetry (Figure 2C). In contrast to anthozoans, bilaterians have many structures that define their bilateriality. For example, bilaterial symmetry is defined in the earthworm by the dorsal and ventral blood vessels, the ventral nerve chord, and the intestine, to name a few (Figure 2D). Given that a limited set of developmental genes are responsible for patterning the body axis of most animals, a better understanding of the evolutionary history of these gene families will contribute greatly to our understanding of this tendency towards axial complexity in early metazoan history.

## Developmental genes in early-diverging phyla

Studies from early-branching metazoans hold much promise towards furthering our understanding of the genetic and developmental basis of morphological change. The first step in this process involves establishing reliable phylogenetic classifications of gene families that highlight orthologous relationships, as well as identifying novel genes within each lineage. The next step entails identifying functional parallels (if they exist) between developmental genes in these early-branching taxa and their corresponding orthologs in Bilateria.

### Hox and homeobox evolution near the base of the metazoa

Studying the homeobox superclass of transcription factors has yielded significant insight towards the goal of understanding basic developmental processes. Homeobox genes play critical roles in development in plants, fungi, and animals. A recent report showed that the common ancestor of bilaterians possessed at least 88 homeobox genes [31]. A subsequent study showed that at least 56 of these homeobox genes were likely present in the cnidarian-bilaterian ancestor [26]. This analysis demonstrates that many important genetic components in the bilaterian developmental toolkit were present in the last common ancestor of cnidarians and bilaterians.

The representation of *Nematostella *homeoboxes within the Hox families was conspicuously deficient when compared to the normal distribution of *Nematostella *to bilaterian genes within multi-family clades [26]. The Hox genes are essential for patterning the primary body axis of bilaterians, and mutations in these genes have homeotic effects on the developing embryo [32]. Evidence from the sequenced genome of the demosponge *Amphimedon *suggests that Hox genes emerged after the metazoan crown ancestor [33]. Furthermore, it seems unlikely that Ctenophores have Hox genes, given that there has been no report of the presence of Hox genes despite two EST sequencing projects [34,35] and several degenerate PCR-based fishing projects [36-38].

Several previous studies have shown that Hox homologues existed in a wide range of cnidarians (reviewed in [39,40]). However, the homeobox survey in *Nematostella *was the first report of a full repertoire of Hox genes from a cnidarian [26]. Subsequent studies have since demonstrated that *Nematostella *has at least seven Hox genes belonging to three distinct families [20,41,42]. In addition, genes from two of these families are organized in a cluster reminiscent of (but distinct in arrangement to) the Hox cluster of bilaterians [20,42]. Furthermore, two additional genes from *Nematostella *were shown to be related to the three ParaHox genes of bilaterians [20,41,42]. These genes are also found in an independent cluster in many bilaterians, and this ParaHox cluster is believed to be a sister cluster to the Hox complex [43] (but see [42] for an alternate theory on the origin of the ParaHox cluster).

If we assume modest and uniform gene loss in the human, *Drosophila*, and *Nematostella *homeobox superfamilies, then the Hox subclass, more than any other subclass of homeobox genes besides Dux (which encode "double homeodomain" proteins), has underwent extensive independent radiation in the bilaterian lineage compared to the cnidarian lineage [26]. A conservative estimate is that the last common ancestor of cnidarians and bilaterians had three Hox and two ParaHox genes that likely radiated into seven Hox and three ParaHox genes in the crown bilaterian ancestor. The Hox gene radiation coincided with a significant increase in axial specificity (compare Figures 2C and 2D). This, combined with the fact that Hox genes likely arose after the divergence of Ctenophora from the rest of Metazoa, is consistent with the hypothesis that complexity and the number of Hox genes in a genome are positively correlated [44-48]. It has previously been observed that within the Bilateria, the level of complexity of a species does not necessarily correlate with the number of Hox genes in its genome [49]. Nevertheless, the pattern seen in these early branching lineages may offer insight into the early evolution of bilateral symmetry and its molecular underpinnings.

## Hox, Wnt, and the evolution of the primary body axis

### Does the Hox system define metazoans?

An influential review by Slack and coworkers hypothesized that Hox genes and their coordinated spatial expression along the primary body axis are metazoan synapomorphies [50]. Early reports of cnidarian Hox genes set off a debate as to whether or not this zootype hypothesis was valid, in light of these new findings [44,51-53]. With the recent release of genomic data from *Nematostella *[54] and other species from early-diverging phyla, there is substantially more information from which to reevaluate the zootype hypothesis.

The evidence suggesting that the last common cnidarian-bilaterian ancestor had a Hox system is consistent with the zootype hypothesis. However, the apparent absence of Hox genes from Ctenophora and Porifera suggests that a Hox system was not in place in the earliest animals. In light of this evidence, the zootype hypothesis must be rejected *sensu stricto*. It is possible that the specification of regional identity along the primary body axis by a set of genes is a synapomorphy of the animal kingdom, and that the Hox genes were not the original set of genes responsible for this specification. If this is the case, then there must have been other genes responsible for patterning the primary body axis of the last common metazoan ancestor. It may be that this ancestral patterning system is still operational in sponges and ctenophores and that traces of the system in cnidarians and bilaterians might perhaps be detected.

### Wnt pathway components in the early-diverging phyla

It has been postulated that the Wnt pathway may be the ancestral metazoan axial patterning system [55]. Several lines of evidence support this hypothesis. Firstly, components of the Wnt pathway have been found in two species of demosponge [9,56] and, a recent study showed the Wnt expression pattern in the demosponge *Amphimedon queenslandica *to be consistent with a role in patterning the primary body axis during development[57]. Furthermore, Wnt signaling is involved in axial patterning in bilaterians. In vertebrates, hemichordates, and echinoderms, Wnt signaling is essential for posterior patterning (reviewed in [58]). In protostomes, there is evidence of expression in the posterior terminus of several insects and the posterior hindgut of polychaete annelids [59]. Similarly, the Dickkopf (Dkk) family of Wnt antagonists are required for formation of anterior structures in vertebrates (reviewed in [60]).

Lastly, phylogenetic and expression analyses from several cnidarians show that Wnt signaling plays a significant role in patterning the cnidarian primary body axis [27,55,61-66]. Genomic data show that there are at least 13 Wnt orthologs in *Nematostella *representing 11 of the 12 bilaterian Wnt subfamilies indicating that the Wnt family had extensively radiated prior to the last common cnidarian-bilaterian ancestor [27,55]. Strikingly, *in situ *hybridization experiments reveal overlapping expression domains of these 13 Wnt genes at the oral end of the developing planula, encompassing roughly 75% of the animal in both germ layers. Furthermore, ectodermal expression of Dkk1/2/4, a gene whose vertebrate orthologs are major antagonists of Wnt ligands, is seen in the aboral region of the *Nematostella *planula, bringing the total Wnt-related signaling coverage of the developing planula close to 100% [27,55].

### Co-option of Hox genes

If a more primitive mechanism responsible for patterning the primary body axis of metazoans than the Hox system exists, then it is likely that the Hox genes were co-opted into this pathway sometime between their origin and the last common ancestor of cnidarians and bilaterians. Recent evidence from both protostomes and deuterostomes suggests that Wnt signaling partitions Hox and ParaHox domains to specify unique cell fates during development [67-71]. Additionally, Wnt genes are known to induce the expression of certain Hox genes under certain conditions in vertebrates [72]. Finally, evidence suggests that Wnt signaling works together with the ParaHox gene Cdx to regulate posterior Hox gene patterning in the mouse embryo (reviewed in [73]). These mounting data suggest that Wnt and Hox genes work together during axial specification.

It is possible, then, that the coordination of these two systems seen in modern-day bilaterians are traces of an ancient co-option event that occurred early in metazoan evolution. If the Hox genes were truly co-opted into the Wnt pathway, it is difficult to say whether the they were co-opted prior to the last common ancestor of cnidarians and bilaterians or subsequently, in the lineage leading to the last common ancestor of bilaterians. The expression patterns of Hox genes in the anthozoan *Nematostella *[42] and the hydrozoan *Eleutheria *[41] show that, while the cnidarian Hox genes are expressed in restricted regions along the primary body axis, the contiguous striped pattern that is seen in the bilaterian Hox genes is not observed. This could be an indication that the co-option process had began prior to the last common ancestor of bilaterians and cnidarians, but became more pronounced later, in the bilaterian lineage.

Speculation on the mechanisms underlying this co-option event might be premature. However, one possibility could be that a single Hox or ParaHox gene, which was high in the Hox/ParaHox cascade, was co-opted into the Wnt network. The Cdx genes (ParaHox) have been shown to be direct targets of Wnt signaling [74,75] and thought to be direct regulators of Hox genes in vertebrates (reviewed in [73,76]). At some point (perhaps prior to the cnidarian-bilaterian ancestor), the ortholog to Cdx could have come under the regulatory control of one or more Wnt genes. As the Hox/ParaHox family expanded through duplication, existing auto-regulatory mechanisms may have been transformed into an axial specifying network controlled initially by the Cdx ParaHox gene, which was under control of a smaller number of Wnt genes. This is only one of many other possible scenarios involving Wnt and/or other axial patterning networks. Nonetheless, these scenarios are testable and, on the surface, seem more likely than a scenario in which the Hox genes produced an independent primary body axis patterning network *de novo*. The initial wave of data from these early branching lineages is not yet at the stage where we can draw more definitive evolutionary scenarios, but they do provide a jumping-off point from which to generate testable hypotheses.

A better understanding of these evolutionary processes will come from more-strongly establishing the relationship between Hox, ParaHox, and Wnt genes in cnidarians, while refining those same relationships in bilaterians. A first step with respect to the Cnidaria would involve establishing the spatial and temporal relationships of the expression of these genes relative to each other. The next step would be to determine the effect of Wnt expression when Hox expression is perturbed and *vice versa*. If the co-option hypothesis holds, these data might help to reveal the progression of this co-option event relative to the cnidarian-bilaterian divergence as well a better understanding of the molecular mechanisms underlying bilaterian complexity and diversity.

## Conclusion

It is becoming clear that combinatorial regulation plays a large part in cell fate specification [68]. A variety of transcription factors and signaling pathways active in a particular cell play a critical role in determining the fate of that cell. During embryogenesis, this combinatorial system is used to impart complexity into the developing embryo. It seems reasonable to postulate that an expansion of gene families involved in cell determination (akin to what is seen in the Wnt and Hox genes during the major transitions in metazoan history) would lead to an increase in cell types and tissue types available to embryogenesis; this would, in turn, lead to more complexity and variety in body plans. Characterizing the dynamics of gene family radiation, gene loss, and co-option is key to understanding these important metazoan transitions. By relating these data with morphological and developmental data in a phylogenetic context, we can begin to understand how changes in ontogeny have led to the burst of morphological diversity that occurred in the early Cambrian.

## Reviewer's comments

### Reviewer 1: Pierre Pontarotti, Université d'Aix Marseille, Marseille, France

Review of the article Hox, Wnt and the evolution of the primary body axis: insights from the early divergent phyla.

This article shed some light on the understanding and the evolution of the bilaterian animals using knowledge from non bilaterian phyla.

Two main important insights come out from this exegesis of the current literature: 1) the revaluation of the zootype hypothesis and 2) the putative role of the Wnt genes in the partitioning of the body axis in urbilateria.

I think that this review article will help experimental design for the "evo/devo" scientific community.

Specific comments:

"Early divergent phyla and the primary body axis" paragraph:

1) The single cell ancestor hypothesis

What really are the evidence that animals descent from several single cell ancestors? Multicellularity occurred several times independently in the history of life, see for example Fungi. I do not understand why the hypothesis of convergent multicellularity should not be considered in the case of animal.

**Authors' response:***The relationship of animals to single-celled eukaryotes is tangential to our thesis, so we have removed the reference to animal origins in the paper. Please also see the response to Comment 2*.

2) The dating of the occurrence of animal multicellularity

I would be careful about the dating; the fact that we cannot witness older multicellular fossil than the one found in the Ediacaran fauna does not mean that they did not exist.

**Authors' response:***We reworded a couple of sentences to make it clear that these dates are only estimates based on published studies. The following sentences were changed:*

*"All animals descended from a single-celled ancestor (most likely a choanofagellate) over 600 million years ago*[4]. *Bilaterian animals radiated during the Cambrian explosion 544–525 million years ago."*

The text now reads:

*"The origin of animals dates back to over 600 million years ago and perhaps much earlier *[4]. *Fossil evidence and molecular evidence suggest bilaterian animals radiated during the Cambrian period some 500–550 million years ago *[5]."

"Graded morphological complexity" paragraph:

The authors wrote: "sponges are much simpler than bilaterians and that cnidarians occupy an intermediate level of axial complexity". What is the objective parameter of complexity. The authors should explain it (even if this is evident for most of our colleague).

**Authors' response:***The following sentence was changed:*

*"It has been recognized previously that in terms of complexity along the primary body axis, sponges are much simpler than bilaterians and that cnidarians occupy an intermediate level of axial complexity *[29]."

The text now reads:

*"It has been recognized previously that in terms of cell types and tissue types along the primary body axis, sponges are much simpler than bilaterians (i.e., sponges lack true tissues and organs) and that cnidarians occupy an intermediate level of axial complexity *[29]."

Concerning the phylogeny of Animal

Do the authors think that Bilaterian and Cnidarians form a monophylogentic group, do they think the hox where co-opted in the most common ancestor of these two phyla? It should be helpful if the authors discuss this possibility.

**Authors' response:**Figure 1*shows the prevailing view that Cnidaria and Bilateria are sister taxa and form a monophyletic group. Most of the ribosomal RNA phylogenies support this relationship as shown in *Table 1.

We've inserted the following into the text to address the second part of this comment:

*"If the Hox genes were truly co-opted into the Wnt pathway, it is difficult to say whether the they were co-opted prior to the last common ancestor of cnidarians and bilaterians or subsequently, in the lineage leading to the last common ancestor of bilaterians. The expression patterns of Hox genes in the anthozoan Nematostella *[42]*and the hydrozoan Eleutheria *[41]*show that, while the cnidarian Hox genes are expressed in restricted regions along the primary body axis, the contiguous striped pattern that is seen in the bilaterian Hox genes is not observed. This could be an indication that the co-option process had began prior to the last common ancestor of bilaterians and cnidarians, but became more pronounced later, in the bilaterian lineage."*

### Reviewer 2: Gáspár Jékely, Max Planck Institute for Developmental Biology, Tübingen, Germany

Reviewed by: Gáspár Jékely, Max Planck Institute for Developmental Biology, Spemannstrasse 35, Tübingen, 72076 Germany, Tel: +49 7071 601 1310, gaspar.jekely@tuebingen.mpg.de

This paper gives a good overview of the early evolution of axial symmetry and the patterning along the AP axis in Metazoa. The paper is well written and I only have a few comments and suggestions for potential improvement.

The authors propose that the Hox system was probably co-opted into the more ancestral Wnt system for patterning along the AP axis. This is an interesting idea and it would be worth spelling it out in a bit more detail. How could it have happened? Did Hox genes intercalate between Wnt signalling and the target genes of Wnt etc.? Why did it allow more precise axial patterning? An extra figure showing the steps of the evolution of the Wnt-Hox axial patterning system in Metazoa would also help (e.g. schematic embryos with the expression domains).

**Authors' response:***We've inserted the following paragraph into the text to address these questions:"Speculation on the mechanisms underlying this co-option event might be premature. However, one possibility could be that a single Hox or ParaHox gene, which was high in the Hox/ParaHox cascade, was co-opted into the Wnt network. The Cdx genes (ParaHox) have been shown to be direct targets of Wnt signaling *[74,75]*and thought to be direct regulators of Hox genes in vertebrates (reviewed in *[73,76]*). At some point (perhaps prior to the cnidarian-bilaterian ancestor), the ortholog to Cdx could have come under the regulatory control of one or more Wnt genes. As the Hox/ParaHox family expanded through duplication, existing auto-regulatory mechanisms may have been transformed into an axial specifying network controlled initially by the Cdx ParaHox gene, which was under control of a smaller number of Wnt genes. This is only one of many other possible scenarios involving Wnt and/or other axial patterning networks. Nonetheless, these scenarios are testable and, on the surface, seem more likely than a scenario in which the Hox genes produced an independent primary body axis patterning network de novo. The initial wave of data from these early branching lineages is not yet at the stage where we can draw more definitive evolutionary scenarios, but they do provide a jumping-off point from which to generate testable hypotheses."*

Wnt expression was also analysed recently in the sponge *Amphimedon queenslandica *(PLoS ONE 2007 vol. 2 pp. e1031) and was found to be expressed at the posterior pole of larvae. This is in agreement with the idea that Wnt signalling plays an ancestral role in patterning the AP axis. These findings should also be discussed.

**Authors' response:***We inserted the following into the text:*

*"...a recent study showed the Wnt expression pattern in the demosponge Amphimedon queenslandica to be consistent with a role in patterning the primary body axis during development*[57]."

### Reviewer 3: L Aravind, Computational Biology Branch, NCBI, NLM, NIH, Bethesda, USA

This reviewer provided no comments for publication.
